# Recent advances in understanding and managing phantom limb pain

**DOI:** 10.12688/f1000research.19355.1

**Published:** 2019-07-23

**Authors:** Andrea Aternali, Joel Katz

**Affiliations:** 1 Department of Psychology, York University, Toronto, Ontario, Canada

**Keywords:** phantom limb pain, cortical reorganization, referred pain, neuropathic pain, central sensitization, assessment, treatment

## Abstract

Post-amputation phantom limb pain (PLP) is highly prevalent and very difficult to treat. The high-prevalence, high-pain intensity levels, and decreased quality of life associated with PLP compel us to explore novel avenues to prevent, manage, and reverse this chronic pain condition. This narrative review focuses on recent advances in the treatment of PLP and reviews evidence of mechanism-based treatments from randomized controlled trials published over the past 5 years. We review recent evidence for the efficacy of targeted muscle reinnervation, repetitive transcranial magnetic stimulation, imaginal phantom limb exercises, mirror therapy, virtual and augmented reality, and eye movement desensitization and reprocessing therapy. The results indicate that not one of the above treatments is consistently better than a control condition. The challenge remains that there is little level 1 evidence of efficacy for PLP treatments and most treatment trials are underpowered (small sample sizes). The lack of efficacy likely speaks to the multiple mechanisms that contribute to PLP both between and within individuals who have sustained an amputation. Research approaches are called for to classify patients according to shared factors and evaluate treatment efficacy within classes. Subgroup analyses examining sex effects are recommended given the clear differences between males and females in pain mechanisms and outcomes. Use of novel data analytical approaches such as growth mixture modeling for multivariate latent classes may help to identify sub-clusters of patients with common outcome trajectories over time.

Effective treatment of phantom limb pain (PLP) is a central issue that continues to confront amputees and their clinicians. The majority of amputees report PLP at some point after limb amputation, and lifetime prevalence estimates are between 50 and 80% ^1– 3^. The pain is typically neuropathic in origin and referred to the missing limb with qualities of sensation such as throbbing, “pins and needles”, shooting, stabbing, and burning. PLP is usually reported within the first week after amputation and generally decreases in severity and frequency over time in most individuals ^4^. It is accompanied by a variety of secondary effects, including depression, impairments in everyday activities, and decreased quality of life ^5^. Over the past 50 years, researchers have explored how PLP can be treated via medication, surgery, therapy, and numerous other approaches. More than 25 treatments for PLP are currently available yet not one is widely accepted or clearly superior to others ^6, 7^. This likely speaks to the multiple mechanisms that contribute to PLP. Treatments typically target a single proposed mechanism, yet amputees can have PLP that arises from multiple mechanisms. Consequently, not one intervention has been found to be consistently effective.

This article presents a narrative review of randomized controlled trials (RCTs) evaluating the efficacy of PLP interventions published over the past 5 years. In the following sections, we review evidence for the efficacy of targeted muscle reinnervation (TMR), repetitive transcranial magnetic stimulation (rTMS), imaginal phantom limb exercises, mirror therapy (MT), virtual and augmented reality, and eye movement desensitization and reprocessing (EMDR) therapy. The Cochrane Collaboration’s tool ^8^ is used to assess risk of bias for the RCTs included in this review. Each included RCT was evaluated according to seven criteria assessing selection, performance, detection, attrition, reporting, and other biases. Table 1 lists the main features of the included articles along with their associated global risk-of-bias rating. The Supplementary Tables 1 to 10 present the detailed risk-of-bias assessments for each included trial for interested readers. Figure 1 summarizes the risk-of-bias assessments across the seven criteria for each included RCT.

**Table 1.** Summary of the literature reviewed in this article investigating the treatment of phantom limb pain.

Authors, year, and sample size	Site (and percentage) of amputation	Reason(s) for amputation and percentages	Mean time (range) since amputation	Treatment groups	Treatment duration	Main outcome measure(s)	Assessments	Findings	Risk of bias
Dumanian *et al*. ^9^ (2019) (n = 28)	UE = 13% LE = 87%	Trauma = 90% Infection = 10%	(Less than 1 year to more than 10 years)	1. Targeted muscle reinnervation 2. Standard neuroma surgery	N/A	Change in NRS worst pain from baseline to 12 months post-operatively for PLP and residual limb pain	Baseline 3 months 6 months 9 months 12 months	No significant between- group differences in worst PLP or residual limb pain 1 year post- surgery	High risk
Malavera *et al*. ^16^ (2016) (n = 54)	LE = 100%	Trauma = 100%	7.8 years	1. Active rTMS 2. Sham rTMS	20 stimuli of 6 seconds each (54- second intervals), 5 days per week for 2 weeks	PLP intensity measured via a visual analogue scale (VAS)	Baseline 15 days post- rTMS 30 days post- rTMS	No significant between- group differences in PLP scores at either follow-up time	Low risk
Brunelli *et al*. ^19^ (2015) (n = 40)	AK = 73% BK = 27%	Dysvascular = 70% Other = 30%	458 days	1. SAIPAN protocol 2. Standard treatment	~1 hour two times per week for 4 weeks	1. PLP intensity, rate, duration, and bother measured via Prosthesis Evaluation Questionnaire (PEQ) and Brief Pain Inventory (BPI) 2. Phantom limb symptom (PLS) intensity, rate, and bother measured via PEQ	Baseline 1 month 2 months	Significant group differences in PLP rate, duration, and bother from PEQ at 2-month follow-up only. No significant differences with BPI. Significant group differences in PLS rate, intensity, and bother at 2-month follow-up only.	High risk
Finn *et al*. ^20^ (2017) (n = 15)	AE = 40% BE = 60%	Trauma = 100%	4.5 months (0.55–24 months)	1. MT 2. Control (covered mirror) 3. Control (mental visualization)	15 minutes 5 days per week for 4 weeks	PLP intensity measured via VAS	Baseline 4 weeks	No between-group comparisons reported for main outcome measure	High risk
Anaforoğlu Külünkoğlu *et al*. ^21^ (2019) (n = 40)	BK = 100%	Trauma = 100%	13.25 months (3–53 months)	1. MT 2. Phantom exercise (PE)	15 minutes of MT daily at home for 4 weeks PE group performed exercises daily with 15 repetitions	1. PLP intensity measured via VAS 2. Quality of life evaluated via SF-36 3. Psychological status measured using BDI	Baseline 4 weeks 3 months 6 months	Significant group difference in VAS PLP severity, BDI scores, and PF, SF, MH, and V subscales of the SF-36 in favour of the MT group at all follow-up times	High risk
Ol *et al*. ^22^ (2018) (n = 45)	BK = 100%	Trauma = 100%	(15–32 years)	1. MT 2. Tactile 3. Combination (mirror and tactile therapy)	5 minutes every morning and night for 4 weeks	PLP intensity measured via VAS	Baseline 5 weeks 3 months after end of treatment	No significant between- group differences in PLP scores at either follow-up time	High risk
Ramadugu *et al*. ^23^ (2017) (n = 60)	AE = 8% BE = 8% AK = 34% BK = 50%	NR	NR	1. MT 2. Control (covered mirror)	15 minutes every day for 4 weeks	PLP intensity measured via VAS and the short form of the McGill Pain Questionnaire	Baseline 4 weeks 8 weeks 12 weeks 16 weeks (20 weeks, control only)	No between-group comparisons reported for main outcome measures	High risk
Tilak *et al*. ^24^ (2016) (n = 26)	UE = 26% LE = 74%	NR	45 days	1. MT 2. Contralateral transcutaneous electrical nerve stimulation	20 minutes every day for 4 days	PLP intensity measured via VAS and Universal Pain Score (UPS)	Baseline 4 days	No significant between- group differences in PLP intensity at the end of treatment	High risk
Rothgangel *et al*. ^25^ (2018) (n = 75)	AK = 61% K = 7% BK = 32%	Trauma = 32% Dysvascular = 40% Tumor = 14% Other = 14%	~35 months	1. MT followed by teletreatment using augmented reality 2. MT followed by self-delivered MT 3. Sensomotor exercises to the intact limb followed by self-delivered exercises	At least 10 30-minute sessions across 4 weeks followed by 6 weeks of self- delivered treatment	PLP frequency, duration, and intensity (measured via NRS)	Baseline 4 weeks 10 weeks 6 months	No significant between- group differences at 4- or 10-week follow-up. At 6-month follow-up, there were significant between- group differences on PLP duration only, in favor of MT followed by self-delivered MT.	High risk
Rostaminejad *et al*. ^26^ (2017) (n = 60)	AK = 40% BK = 60%	Diabetes = 45% Trauma = 50% Cancer = 5%	(2–38 months)	1. EMDR 2. Routine care	12 one-hour sessions over 1 month	PLP intensity measured via the subjective units of distress scale and pain rating scale	Baseline 1 month 24 months	No between-group comparisons reported for main outcome measures	High risk

Results from interventions using targeted muscle reinnervation, repetitive transcranial magnetic stimulation (rTMS), mirror therapy (MT), augmented reality, and eye movement desensitization and repossessing (EMDR) therapy are summarized above. For each study, the site, reason, and mean time since amputation are shown. Treatment groups and duration as well as main outcome measures, times of assessment, and major findings are included. The final column describes the article’s global risk-of-bias rating as assessed by the Cochrane Collaboration’s tool ^8^. UE and LE refer to upper and lower extremity amputations, respectively. AK, BK, AE, and BE refer to amputations performed above (A) and below (B) the knee (K) and elbow (E) joint. NR denotes information that was not reported by the author. NRS refers to the numeric 0–10 rating scale. BDI, Beck Depression Inventory; MH, mental health; N/A, not applicable; PF, physical functioning; PLP, phantom limb pain; SAIPAN, Santa Lucia Alleviation Intervention for Phantom in Amputees’ Neurorehabilitation; SF, social functioning; SF-36, 36-Item Short Form Survey; V, vitality.

**Figure 1.**
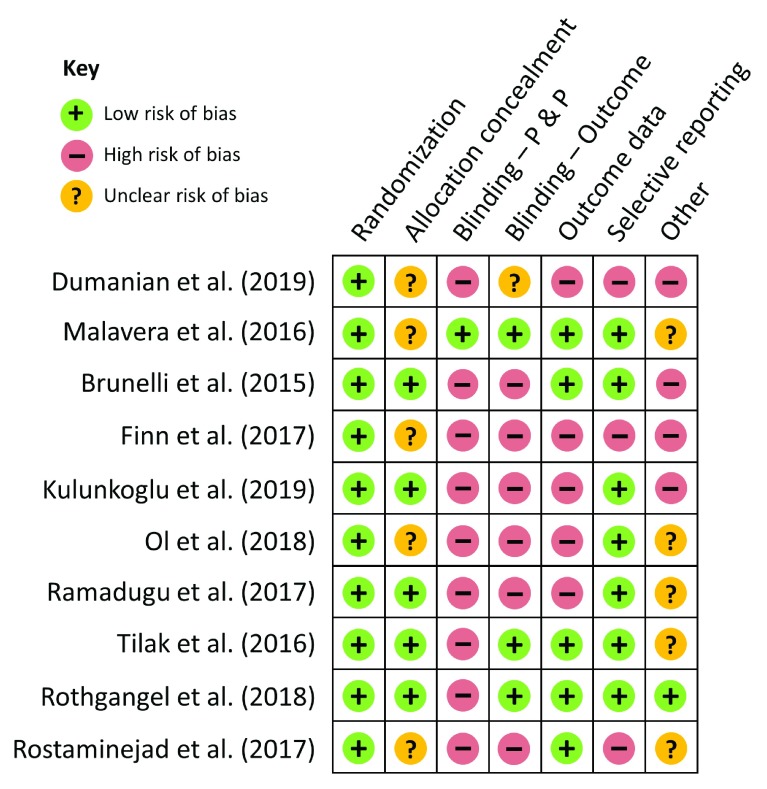
Risk-of-bias ^8^ assessments for studies presented in the review of recent randomized controlled trials exploring treatment of phantom limb pain. Randomization: randomization sequence generation; Blinding – P & P: blinding of participants and personnel; Blinding – Outcome: blinding of outcome assessment; Outcome data: incomplete outcome data.

## Targeted muscle reinnervation

Proposed mechanisms underlying PLP have traditionally been classified according to the level of the nervous system: peripheral or central. Peripheral mechanisms include activation of residual limb neuromas by mechanical stimulation, circulating catecholamines, pro-inflammatory immunological factors, and other pronociceptive neurochemicals. TMR is a relatively recent technique that involves surgically re-routing and coapting the distal aspect of a transected nerve to a motor nerve that innervates an adjacent muscle. Over time, the coapted residual nerve and motor nerve fasten together and the new combined nerve reinnervates the muscle ^9^. The mechanism by which TMR is believed to reduce PLP is not entirely clear, yet researchers have suggested restoration of physiological continuity and function ^10^, neuroma prevention ^11^, and upstream effects on cortical reorganization ^9^. Recent research has suggested that, though originally proposed to facilitate use and control of myoelectric prostheses, TMR may also be an effective way to reduce PLP ^10– 13^.

A recent RCT examined the efficacy of TMR for PLP among 28 unilateral and bilateral upper or lower limb amputees with chronic PLP (30 limbs treated) ^9^. Time since amputation ranged from less than 1 year to more than 10 years. TMR was compared to standard treatment involving excising the neuroma and burying the remaining nerve into neighboring muscle. Worst, best, and current levels of phantom and residual limb pain in the past 24 hours were assessed pre-operatively and post-operatively at 3-month intervals for 1 year. Secondary outcomes, including pain behaviour, pain intensity, and pain interference assessments of the Patient-Reported Outcomes Measurement Information System (PROMIS), were also completed at these assessments. One year after surgery, significant between-group differences were not found in worst PLP intensity, worst residual limb pain intensity, or the three PROMIS pain scales ^9^. However, an analysis using all available data (regardless of whether the last assessment was at the 1-year time point) showed that worst PLP change scores at the final assessment were significantly greater in TMR than standard treatment, indicating greater reduction in PLP for the former group ^9^. Higher baseline pain scores for the intervention group may explain this significant result. Taken together, the results do not support the efficacy of TMR for PLP. Future research should compare TMR with a less invasive but putatively equally efficacious treatment such as non-invasive brain stimulation ^14^.

## Repetitive transcranial magnetic stimulation

Maladaptive cortical re-organization is one of the central neural mechanisms thought to underlie PLP. It has been proposed that, after amputation, cortical areas that once represented the amputated extremity become reoccupied by adjacent zones in the primary somatosensory and motor cortex corresponding to other body parts ^6, 15^. Imagined movement of the phantom extremity is accompanied by brain activity in regions corresponding to not only the lost extremity but also the adjacent body part. PLP has been shown to be more intense among individuals for whom a greater degree of maladaptive cortical remapping has taken place ^6^. rTMS has been explored for its potential in preventing maladaptive sensorimotor cortical remapping and in reducing PLP ^16^. Targeting the somatosensory and motor cortex using a magnetic pulse emitted by the rTMS coil has been proposed to activate descending inhibitory pathways to the thalamus, thereby modulating subsequent ascending nociceptive signals and reducing PLP ^16^.

rTMS was evaluated in an RCT of 54 unilateral trauma-related lower limb amputees ^16^. The mean time since amputation was 7.8 years. Participants were randomly assigned to receive active rTMS or sham rTMS for 20 minutes five times per week over 2 weeks. The control condition was exposed to a sham coil that did not emit a magnetic pulse or induce a tactile sensation on the scalp. For the active rTMS group, the authors targeted the hand area of motor cortex contralateral to the amputated leg, citing evidence of efficacy for this stimulation site in past studies regardless of anatomic location of the pain ^17, 18^. PLP was evaluated daily for 1 week before the start of treatment and 15 and 30 days after the last treatment. Levels of depression and anxiety were also measured at each of these time points. Between-group differences in PLP scores and levels of depression and anxiety were not significant at either follow-up time ^16^. However, PLP scores at both follow-up times were significantly lower than at baseline for the active group but not the sham group. Fifteen days after treatment, there was a significantly greater percentage reduction in PLP in the group that received active versus sham rTMS; however, this was no longer significant 30 days after treatment. rTMS of the motor cortex does not appear to reduce PLP in lower extremity amputees to a greater extent than a sham control condition when the hand area, rather than the foot area, is stimulated. Future studies may prove more effective by matching the rTMS stimulation site in motor cortex contralateral to the extremity amputated.

## Imaginal phantom limb exercises

Cortical reorganization has also been proposed to occur as a result of mental imagery, including engaging in phantom limb exercises (that is, active imaginal efforts to move the phantom), under the assumption that the neural pathways involved in performing actual movements are activated when using one’s imagination to move the phantom extremity. Initial studies of phantom limb exercises show promise in reducing PLP ^27, 28^.

In a 2015 study, unilateral lower limb amputees were randomly assigned to one of two groups to receive progressive muscle relaxation, mental imagery, and phantom exercises (n = 27) or residual limb exercises (n = 24) ^19^. The mean reported time since amputation was 458 days, and the majority of amputations were due to diabetes and peripheral vascular disease. The treatment group received a 50-minute combined training session in progressive muscle relaxation, mental imagery, and phantom exercises twice per week for 4 weeks. Phantom exercises involved imagining moving the phantom limb and then attempting to perform these movements. The control group received the same amount of treatment involving exercising their residual limb. In addition, both groups participated in a rehabilitation program involving occupational therapy and prosthesis training, which took place twice per day for 4 weeks. Items from the Prosthesis Evaluation Questionnaire (PEQ) and Brief Pain Inventory (BPI) measuring pain and bodily sensations were collected at baseline, at the end of treatment, and 1 month after treatment. Owing to participant attrition, only 20 participants from each group were included in the final analysis. At the end of treatment, the two groups did not differ significantly on either the PEQ or BPI items ^19^. At the 1-month follow-up, the treatment group had significantly lower pain intensity scores on the BPI worst and average pain items and significantly lower scores on the PLP rate, intensity, and bother items of the PEQ compared with the control group ^19^. Although preliminary evidence seems promising, the authors did not adjust the type 1 error rate for multiple comparisons. Moreover, the absence of between-group differences in PEQ and BPI outcomes at the end of treatment is puzzling and raises the possibility that some factor unrelated to treatment accounts for the significant effects at the 1-month follow-up. Furthermore, the researchers did not measure the amputees’ self-report of their ability to perform the phantom exercises or the extent to which they engaged in these movements. This is an important factor to measure given that self-reported motor control is a predictor of PLP severity ^29^. More research is needed on the effects of combined progressive muscle relaxation, mental imagery, and phantom exercises using larger sample sizes and better measures.

## Mirror therapy

It has been suggested that PLP may be especially difficult to treat because of the absence of tactile and visual feedback from the limb ^30^. The role of the visual and tactile modality is especially important since they provide important information involving exteroceptive sensibility. Lower limb amputees frequently report that it was not until they looked under the bed sheets and reached out to touch the limb that they realized it had been cut off. When there is a discrepancy or contradiction between incoming information from different modalities or when a state of uncertainty exists based upon somatosensory input alone, additional information is sought via these modalities, which usually determine the perceptual experience. Amputation not only results in the loss of afferent input/feedback from the amputated limb but also produces a loss of visual and tactile information related to the limb. The central influences that normally inhibit pain may be further reduced by the absence of information from these external sources that might otherwise confirm or disconfirm the perception of pain arising from the periphery (for example, a phantom limb in a painful position or a “crawling” sensation on the skin) ^30^. Thus, some forms of PLP may arise, in part, from a mechanism involving a release from inhibitory control (that is, disinhibition).

Self-touch of a painful area can gate pain signals from reaching the brain, therefore minimizing the pain experience ^31^. Looking at one’s own body has also been shown to reduce pain intensity and neural responses to painful stimuli compared with viewing a neutral object ^32^. Research on reducing PLP has focused on restoring this lack of sensory feedback. MT is a long-standing treatment for PLP ^33^ and is thought to reduce PLP by restoring normal somatosensory and visual inputs to associated brain structures, although the precise mechanisms by which this occurs are not well understood ^20^. A recent neuroimaging study of lower limb amputees with PLP found enhanced responsiveness to viewing images of feet (but not hands) in the foot area of sensorimotor cortex contralateral to the amputated limb as well as in posterior parietal cortex ^34^. Both PLP intensity and the increased visual responsiveness were abolished after 4 weeks of MT. These results are consistent with the “PLP as disinhibition” hypothesis ^30^ described above whereby seeing the limb (via MT) re-establishes, in somatosensory and parietal structures, the normal inhibitory control processes which were lost because of amputation (that is, visual deafferentation). The restoration, via the visual modality, of inhibitory control over cells in these and other brain regions reduces abnormal brain activity, which contributed to increased levels of PLP, and thereby reduces pain.

Five RCTs examining the efficacy of MT for PLP have been published over the past 5 years ^20, 21– 24^. Sample sizes range from a total of 15 ^20^ to 60 ^23^. Two studies ^21, 22^ recruited unilateral lower extremity amputees only (n = 85), one study ^20^ recruited unilateral upper limb amputees only (n = 15), and the remaining two studies ^23, 24^ recruited both unilateral lower (n = 60) and unilateral upper (n = 17) extremity amputees. Time since amputation ranged from less than 1 month ^20^ to 32 years ^22^; one study ^23^ did not report time since amputation. MT was compared with sensorimotor exercises with or without a covered mirror ^20, 21, 23^, tactile therapy ^22^, or contralateral transcutaneous electrical nerve stimulation ^24^, a treatment that has been shown to be effective for PLP ^35– 37^. Parameters of the MT intervention ranged from a low of a single 20-minute session daily for 4 consecutive days ^24^ to 5-minute sessions twice per day for 4 weeks ^22^ to a high of one 15-minute session daily for 4 weeks ^20, 21, 23^. Participants were instructed to move both the intact and phantom limb synchronously during MT while viewing the reflected image of the intact limb (that is, the phantom) in the mirror ^20, 21– 23^. One study did not specify what participants were instructed to do regarding phantom limb exercises ^24^. PLP was assessed at various times, including pre-treatment baseline and immediately post-treatment for all studies and up to 3 ^22^, 4 ^23^, or 6 ^21^ months post-treatment. Of the five studies, only one showed significantly lower PLP intensity scores in favour of MT up to 6 months after treatment ^21^. The remaining studies either did not report a between-group test of PLP intensity ^20, 23^ or failed to show a significant benefit of MT on any measure of PLP at the end of treatment ^22, 24^. Taken together, the results of the most recent studies evaluating the efficacy of MT for PLP are not promising. Overall, MT does not appear to reduce PLP to a greater degree than control or other known treatments.

## Virtual and augmented reality

Virtual and augmented reality interventions have recently emerged as novel approaches to treating PLP. Virtual reality involves completely immersing an individual in a virtual world, whereas augmented reality adds digital elements, such as the missing limb, to a real environment. These interventions represent a “high-tech” alternative to traditional MT ^38^ as they allow amputees to move their intact and phantom limbs independently while seeing their phantom limb integrated into, and interacting with, the surrounding setting ^39^. They also represent a more engaging form of treatment which may increase adherence ^39^. Although virtual and augmented reality interventions have gained popularity, quality evidence does not exist to support its efficacy ^40^. Existing studies are typically underpowered and lack comparison groups.

In spite of the excitement and popularity surrounding virtual and augmented reality for treatment of PLP, only one RCT using augmented reality has been published in the past 5 years ^25^. Seventy-five unilateral lower limb amputees with a median time since amputation of about 3.5 years were randomly assigned to one of three interventions. The first group completed 4 weeks of MT followed by 6 weeks of teletreatment involving augmented reality and digital exercise programs. The second group underwent 4 weeks of MT followed by 6 weeks of self-delivered MT (traditional MT group). The third group received 4 weeks of sensorimotor exercises to the unamputated limb and 6 weeks of self-delivered exercises (control group). Each group received at least 10 30-minute sessions of their respective intervention across the initial 4 weeks. Participants assigned to the MT intervention were instructed to perform exercises using their intact limb in front of a mirror. Only once they perceived voluntary and pain-free movements of their phantom limb were they asked to engage in phantom exercises. PLP ratings of intensity, frequency, and duration were collected at baseline and 4, 10, and 24 weeks later. At the 4- and 10-week follow-ups, the three groups did not differ significantly on any of the PLP measures. At the 6-month follow-up, the duration of PLP episodes was significantly shorter in the traditional MT group in comparison with the control and teletreatment groups. In contrast, 6-month average PLP intensity and 6-month PLP frequency did not differ between the groups; moreover, not one of the three PLP outcome measures showed a significant between-group difference immediately after treatment (4 weeks) or at the 10-week follow-up. This raises the possibility that the 6-month outcome was due to factors other than traditional MT. The results do not favour MT or augmented reality as a viable treatment for PLP. Further research is needed to determine whether virtual and augmented reality are effective in the treatment of PLP.

## Eye movement desensitization and reprocessing

Researchers have also explored treatments that target psychological mechanisms maintaining PLP, such as painful and traumatic memories. One such intervention is EMDR therapy. EMDR is thought to reduce PLP by emotionally processing “painful memories” that are proposed to maintain PLP ^26^. EMDR therapy was compared with a control condition in an RCT of 60 unilateral lower limb amputees ^26^. The reason for amputation was trauma (50%), diabetes-related complications (45%), or cancer (5%). Time since amputation ranged from 2 to 38 months. The control group received routine care while the experimental group underwent 12 one-hour sessions of EMDR administered by trained psychologists over 1 month. Average PLP ratings were collected at baseline, at the end of treatment, and 24 months later. The authors did not report the results of a between-group analysis at any point in time. PLP intensity in the EMDR group was significantly lower than baseline after treatment and at the 24-month follow-up. In contrast, PLP intensity in the control group remained consistently high across the study period. Further studies are needed to determine whether EMDR effectively reduces PLP.

## Summary and Conclusions

The results of this review do not support the efficacy of any of the treatments described for PLP, including TMR, rTMS, imaginal phantom limb exercises, MT, augmented reality, or EMDR therapy. The multiple mechanisms underlying PLP have made it difficult to treat, and one specific treatment that targets multiple mechanisms of PLP has yet to evolve. Moreover, the published literature does not assess the putative mechanism(s) causing PLP in individuals recruited into clinical trials and so it is not surprising that, on average, PLP in the treatment group does not differ from that in the control group. This may explain why many of the treatments available are ineffective. Many of the studies reviewed have small sample sizes with short follow-up periods ( Table 1). Little has changed in the more than 20 years since the recommendation for a rational approach to assessment and management of PLP ^41, 42^. The field continues to lack a mechanism-based method of classifying amputees.

## Recommendations for future studies

This review of RCTs conducted over the past 5 years has not demonstrated consistent evidence for a given intervention. The data in Supplementary Tables 1 to 10 and Figure 1 show that the risk of bias is high for most of the studies included in this review, thus raising questions about the studies’ internal validity and quality. These data highlight the need to develop guidelines on how to improve future PLP treatment research. We suggest that in addition to improving the methodological quality of studies by adhering to the most recent Consolidated Standards of Reporting Trials (CONSORT) statement (http://www.consort-statement.org), the following methodological improvements are required: larger sample sizes, long-term follow-ups, and limiting inclusion criteria for any given study to minimize participant heterogeneity. We recommend limiting recruitment for RCTs to upper or lower extremity amputations, trauma-related or vascular disease-related amputations, and short or long time since amputation. Moreover, given the importance of sex differences in the field of pain ^43, 44^, we strongly recommend that subgroup analyses look separately at female and male amputees. Finally, the use of average pain scores as the best measure of treatment efficacy has been criticized on empirical and theoretical grounds ^45^. Adopting novel data analytical approaches such as growth mixture modeling for multivariate latent classes may help to identify sub-clusters of patients with common outcome trajectories over time.
