# Supplementary material for: Recent advances in understanding and managing phantom limb pain
Source: F1000Res. 2019 Jul 23;8:F1000 Faculty Rev-1167. [Version 1] doi: 10.12688/f1000research.19355.1 (PMC6652103; doi:10.12688/f1000research.19355.1)
Supplement: Supplementary file 1 [file f1000research-8-21216-s0000.tgz › 9546e6ad-c2ef-4995-b3ba-6d4af0978f5c_Katz_Supplementary_Tables_Final.pdf]

Supplementary Table 1. Risk of bias assessment of Dumanian *et al*. (2018)[1]

**Bias**	**Author's Judgment**	**Support for Judgment**
Random sequence generation (selection bias)	Low risk	Participants were randomized using a random number generator
Allocation concealment (selection bias)	Unclear risk	Description not provided of how/whether the allocation sequence was concealed
Blinding of participants and researchers (performance bias)	High risk	Single-blind
Blinding of outcome assessment (detection bias)	Unclear risk	No description of whether researchers were blind at outcome assessments
Incomplete outcome data (attrition bias)	High risk	Eighty-five participants were screened, 28 of whom participated. Authors did not provide details as to why not all individuals participated. Loss to follow-up was not disclosed. Fourteen participants were included from each group in the final analysis.
Selective reporting (reporting bias)	High risk	While all prespecified outcomes were reported, the authors also conducted and interpreted analyses using all available data, regardless of whether the last assessment was at the same time point for all participants.
Other bias	High risk	The intervention group had higher baseline pain scores in comparison to the control group.

Supplementary Table 2. Risk of bias assessment of Malavera *et al.* (2016)[2]

**Bias**	**Author's Judgment**	**Support for Judgment**
Random sequence generation (selection bias)	Low risk	Quote: "A computer-generated randomization method with a permuted block size of 6 was used to allocate subjects to the sham or active Repetitive transcranial magnetic stimulation (rTMS) interventions"
Allocation concealment (selection bias)	Unclear risk	Description not provided of how/whether the allocation sequence was concealed
Blinding of participants and researchers (performance bias)	Low risk	Double-blind
Blinding of outcome assessment (detection bias)	Low risk	Quote: "All evaluations were performed by an investigator blinded to treatment allocation"
Incomplete outcome data (attrition bias)	Low risk	Loss to follow-up was disclosed. Out of the 97 participants assessed for eligibility, 54 were randomized and participated in the study. At the first follow-up, 1 participant from each group was lost due to attrition. At the second follow-up, 2 participants from each group were lost for the same reason. Not one participant was excluded from the final analysis (n = 27/group)
Selective reporting (reporting bias)	Low risk	All prespecified outcomes were reported
Other bias	Unclear risk	rTMS of the motor cortex targeted the hand region rather than the foot region. It is unclear how this may bias the results obtained

Supplementary Table 3. Risk of bias assessment of Brunelli *et al*. (2015)[3]

Bias	Author's Judgment	Support for Judgment
Random sequence generation (selection bias)	Low risk	Quote: "the participants were randomly assigned to 2 groups in a 1:1 ratio, on the basis of a computer-generated list, by an investigator not involved in the evaluations"
Allocation concealment (selection bias)	Low risk	Quote: "The group allocation list was concealed from both physical therapists and subjects"
Blinding of participants and researchers (performance bias)	High risk	Open label
Blinding of outcome assessment (detection bias)	High risk	Open label
Incomplete outcome data (attrition bias)	Low risk	One hundred seven individuals were assessed for eligibility. Fifty-six participants were excluded for reasons described by the authors. Of the remaining 51 participants, 15 were lost at various follow-up times. Forty (20 participants per group) were included in the final analysis
Selective reporting (reporting bias)	Low risk	All prespecified outcomes were reported
Other bias	High risk	Authors did not adjust the Type I error rate for multiple comparisons or measure participants' self-report of their ability to perform phantom exercises which may highly bias the obtained results.

Supplementary Table 4. Risk of bias assessment of Finn *et al*. (2017)[4]

Bias	Author's Judgment	Support for Judgment
Random sequence generation (selection bias)	Low risk	Quote: "Using a computer-generated number, participants were randomly assigned to three groups"
Allocation concealment (selection bias)	Unclear risk	Description not provided of how/whether the allocation sequence was concealed
Blinding of participants and researchers (performance bias)	High risk	Open label
Blinding of outcome assessment (detection bias)	High risk	Open label
Incomplete outcome data (attrition bias)	High risk	Twenty participants were assessed for eligibility. Five withdrew before baseline. Reasons for withdrawal were not provided by the authors. Fifteen participated and were allocated to the intervention and control groups. No participants were excluded from the final analysis
Selective reporting (reporting bias)	High risk	Between group comparisons were not reported for the main outcome measure
Other bias	High risk	Fifteen participants were unevenly distributed across the 3 groups such that 9 were allocated to the intervention and 3 were allocated to both control groups. Low power risks biased results

Supplementary Table 5. Risk of bias assessment of Kulunkoglu *et al*. (2019)[5]

**Bias**	**Author's Judgment**	**Support for Judgment**
Random sequence generation (selection bias)	Low risk	Quote: "amputees were assigned to one group using the closed envelop randomization technique"
Allocation concealment (selection bias)	Low risk	Group allocation was concealed in closed envelopes
Blinding of participants and researchers (performance bias)	High risk	Open label
Blinding of outcome assessment (detection bias)	High risk	Open label
Incomplete outcome data (attrition bias)	High risk	The authors did not disclose the number of participants screened for eligibility. Forty participants were assigned into two groups (20 subjects per group). Explanations for attrition or exclusions from the final analysis were not provided
Selective reporting (reporting bias)	Low risk	All prespecified outcomes were reported
Other bias	High risk	The number and duration of sessions for the intervention group was greater than that of the active control group. This may have biased the results in favor of the treatment group

Supplementary Table 6. Risk of bias assessment of Ol *et al*. (2018)[6]

**Bias**	**Author's Judgment**	**Support for Judgment**
Random sequence generation (selection bias)	Low risk	Quote: "computer-generated random numbers were used for simple randomization"
Allocation concealment (selection bias)	Unclear risk	Description not provided of how/whether the allocation sequence was concealed
Blinding of participants and researchers (performance bias)	High risk	Open label
Blinding of outcome assessment (detection bias)	High risk	Open label
Incomplete outcome data (attrition bias)	High risk	The authors did not disclose the number of participants screened for eligibility. Forty-five participants were assigned into three groups (15 subjects per group). Only one participant left the study for reasons unrelated to the study
Selective reporting (reporting bias)	Low risk	All prespecified outcomes were reported
Other bias	Unclear risk	The authors made deviations from the trial protocol. Two participants who should have been denied participation according to exclusion criteria were permitted to participate. This may have biased the results.

Supplementary Table 7. Risk of bias assessment of Ramadugu *et al*. (2017)[7]

**Bias**	**Author's Judgment**	**Support for Judgment**
Random sequence generation (selection bias)	Low risk	Participants were randomly assigned to a group using a random number table
Allocation concealment (selection bias)	Low risk	In order to conceal group allocation, assigned treatment information was kept in a sealed envelope.
Blinding of participants and researchers (performance bias)	High risk	Open label
Blinding of outcome assessment (detection bias)	High risk	Open label
Incomplete outcome data (attrition bias)	High risk	The authors did not disclose the number of participants screened for eligibility. Of the 64 participants recruited, 4 participants left the study for reasons described by the authors. Sixty participants were included in the final analysis (32 in the test group, 28 in the control group).
Selective reporting (reporting bias)	Low risk	All prespecified outcomes were reported
Other bias	Unclear risk	Reason and time since amputation for the study sample were not reported by the authors. This may have biased the obtained results

Supplementary Table 8. Risk of bias assessment of Tilak *et al*. (2015)[8]

**Bias**	**Author's Judgment**	**Support for Judgment**
Random sequence generation (selection bias)	Low risk	Quote: "A computer generated simple randomization sequence was carried out"
Allocation concealment (selection bias)	Low risk	Randomization allocation was concealed using opaque envelopes and was performed by a researcher not directly involved in the treatment or assessment of subjects
Blinding of participants and researchers (performance bias)	High risk	Open label
Blinding of outcome assessment (detection bias)	Low risk	Subjects were evaluated at baseline and at follow-up by an individual blinded to treatment allocation.
Incomplete outcome data (attrition bias)	Low risk	Thirty-two participants were screened for eligibility. Six were excluded for not meeting criteria or declining to participate. The remaining 26 participants were randomized into two groups (13 participants per group). One participant in the treatment group dropped out of the study.
Selective reporting (reporting bias)	Low risk	All prespecified outcomes were reported
Other bias	Unclear risk	Reason for amputation was not reported by the authors. This may have biased the obtained results

Supplementary Table 9. Risk of bias assessment of Rothgangel *et al*. (2018)[9]

Bias	Author's Judgment	Support for Judgment
Random sequence generation (selection bias)	Low risk	Participants were block-randomized into groups using block sizes of six
Allocation concealment (selection bias)	Low risk	The principal investigator electronically concealed group assignment. They were the only who could break the randomization code.
Blinding of participants and researchers (performance bias)	High risk	Single-blind
Blinding of outcome assessment (detection bias)	Low risk	Quote: "the assistant asked patients not to reveal the assigned treatment during the measurement"
Incomplete outcome data (attrition bias)	Low risk	Two hundred sixty-nine participants were assessed for eligibility. For various reasons, 194 were deemed ineligible. Seventy-five participants enrolled and were allocated to one of the three groups. The first group had 26 participants, the second had 25, and the third had 24. In total, 13 did not complete the study for various reasons described by the authors. Sixty-four were included in the analysis at the final follow-up time
Selective reporting (reporting bias)	Low risk	All prespecified outcomes were reported
Other bias	Low risk	The researchers used a heterogenous sample with not matched for gender, reason for amputation, telescoping, and perceived range of motion of the phantom limb. Mixed model analyses were conducted to correct for these differences. This may have biased the obtained results

Supplementary Table 10. Risk of bias assessment of Rostaminejad *et al*. (2017)[10]

Bias	Author's Judgment	Support for Judgment
Random sequence generation (selection bias)	Low risk	Quote: "The samples were randomly based on parallel and random allocation of block randomization divided into two experimental and control groups"
Allocation concealment (selection bias)	Unclear risk	Description not provided of how/whether the allocation sequence was concealed
Blinding of participants and researchers (performance bias)	High risk	Open label
Blinding of outcome assessment (detection bias)	High risk	Open label
Incomplete outcome data (attrition bias)	Low risk	Eighty-five participants were assessed for eligibility. Twenty-five were excluded for not meeting inclusion criteria (n = 23) or refusing to participate (n = 2). The remaining 60 were randomized into two groups of 30. All participants were included in the final analysis
Selective reporting (reporting bias)	High risk	Between group comparisons were not reported for the main outcome measures
Other bias	Unclear risk	Time since amputation ranged from 2 to 38 months. Given the intervention is focused on traumatic memories, treatment may not have had the same effect on participants who more recently experienced the trauma. This may have biased the results

References

1. Dumanian GA, Potter BK, Mioton LM, Ko JH, Cheesborough JE, Souza JM, Ertl WJ, Tintle SM, Nanos GP, Valerio IL, Kuiken TA, Apkarian AV, Porter K, Jordan SW. **Targeted Muscle Reinnervation Treats Neuroma and Phantom Pain in Major Limb Amputees: A Randomized Clinical Trial.** *Ann Surg* 2018. doi:10.1097/SLA.0000000000003088.

2. Malavera A, Silva FA, Fregni F, Carrillo S, Garcia RG. **Repetitive Transcranial Magnetic Stimulation for Phantom Limb Pain in Land Mine Victims: A Double-Blinded, Randomized, Sham-Controlled Trial.** *J Pain* 2016;**17**:911–8. doi:10.1016/j.jpain.2016.05.003.

3. Brunelli S, Morone G, Iosa M, Ciotti C, Giorgi R de, Foti C, Traballesi M. **Efficacy of Progressive Muscle Relaxation, Mental Imagery, and Phantom Exercise Training on Phantom Limb: A Randomized Controlled Trial.** *Arch Phys Med Rehabil* 2015;**96**:181–7. doi:10.1016/j.apmr.2014.09.035.

4. Finn SB, Perry BN, Clasing JE, Walters LS, Jarzombek SL, Curran S, Rouhanian M, Keszler MS, Hussey-Andersen LK, Weeks SR, Pasquina PF, Tsao JW. **A Randomized, Controlled Trial of Mirror Therapy for Upper Extremity Phantom Limb Pain in Male Amputees.** *Front Neurol* 2017;**8**:1910. doi:10.3389/fneur.2017.00267.

5. Anaforoğlu Külünkoğlu B, Erbahçeci F, ALKAN A. **A comparison of the effects of mirror therapy and phantom exercises on phantom limb pain.** *Turk J Med Sci* 2019;**49**:101–9. doi:10.3906/sag-1712-166.

6. Ol HS, van Heng Y, Danielsson L, Husum H. **Mirror therapy for phantom limb and stump pain: A randomized controlled clinical trial in landmine amputees in Cambodia.** *Scandinavian Journal of Pain* 2018;**18**:603–10. doi:10.1515/sjpain-2018-0042.

7. Ramadugu S, Nagabushnam SC, Katuwal N, Chatterjee K. **Intervention for phantom limb pain: A randomized single crossover study of mirror therapy.** *Indian J Psychiatry* 2017;**59**:457–64. doi:10.4103/psychiatry.IndianJPsychiatry_259_16.

8. Tilak M, Isaac SA, Fletcher J, Vasanthan LT, Subbaiah RS, Babu A, Bhide R, Tharion G. **Mirror Therapy and Transcutaneous Electrical Nerve Stimulation for Management of Phantom Limb Pain in Amputees - A Single Blinded Randomized Controlled Trial.** *Physiother. Res. Int.* 2016;**21**:109–15. doi:10.1002/pri.1626.

9. Rothgangel A, Braun S, Winkens B, Beurskens A, Smeets R. **Traditional and augmented reality mirror therapy for patients with chronic phantom limb pain (PACT study): Results of a three-group, multicentre single-blind randomized controlled trial.** *Clin Rehabil* 2018;**32**:1591–608. doi:10.1177/0269215518785948.

10. Rostaminejad A, Behnammoghadam M, Rostaminejad M, Behnammoghadam Z, Bashti S. **Efficacy of eye movement desensitization and reprocessing on the phantom limb pain of patients with amputations within a 24-month follow-up.** *International Journal of Rehabilitation Research* 2017;**40**:209–14. doi:10.1097/MRR.0000000000000227.
